# Tocilizumab for relapsing and remitting giant cell arteritis: a case series

**DOI:** 10.1186/s13256-022-03625-y

**Published:** 2022-10-27

**Authors:** Pratyasha Saha, Denesh Srikantharajah, Arvind Kaul, Nidhi Sofat

**Affiliations:** 1 Institute for Infection & Immunity, St George’s University of London, Cranmer Terrace, London, SW17 0RE UK; 2 St George’s University Hospitals NHS Foundation Trust, Blackshaw Road, London, SW17 0QT UK

**Keywords:** Giant cell arteritis, Pathway, Tocilizumab, Corticosteroids, Vasculitis, Case series

## Abstract

**Background:**

Giant cell arteritis is a large vessel vasculitis of the arteries in the head and neck. The mainstay of management is with high-dose corticosteroids, and patients often face difficulties stopping or reducing steroids without recurrence of symptoms. Corticosteroids are well established to have numerous associated side effects, including osteoporosis, weight gain, and diabetes. Therefore, when tocilizumab was approved for up to 1 year for cases of relapsing or refractory giant cell arteritis by the National Institute of Health and Care Excellence (NICE) in April 2018, this offered an opportunity to benefit from new funding and to reduce steroid burden.

**Case presentation:**

This case series describes the impact of the establishment of a new hub and spoke referral pathway for the use of tocilizumab in refractory or relapsing giant cell arteritis, with case examples from consecutive patients who accessed the funding between August 2018 and April 2021. A total of 16 patients were identified: 11 female and 5 male, with an average age of 72.4 (range 61–82) years, with a majority of 11 ethnically white. The central assessing hub is St George’s University Hospitals NHS Foundation Trust Hospital, serving a population of 1.3 million in the south of England. This is the first large case series looking into the impact of the establishment of a regional clinical pathway for the new tocilizumab funding.

**Conclusions:**

The case series demonstrates that the use of tocilizumab has reduced both the duration and the dose of corticosteroids in these 16 cases (mean prednisolone reduction 20.4 mg: 95% CI 13.0–27.8 mg), with 50% of patients continuing on tocilizumab after the initial 12 month funding period. The disease course, patterns of response, and maintenance of remission are discussed, and we describe the benefits of replicating this hub and spoke tocilizumab pathway in other centers.

## Background

Giant cell arteritis (GCA) is a rapidly progressive large vessel vasculitis, where patients present with headache, visual loss, jaw pain, fatigue, and myalgia [1]. It is the most common vasculitis, and is often encountered initially in the emergency department or by general practitioners, where it constitutes a medical emergency on presentation. Demographically the average age of onset for GCA is 70 years of age, and it is seven times more common in Caucasians, three times more common in women than men [2], and has an incidence of 19 per 100,000 people above the age of 50 years [3].

The current criteria for diagnosing GCA has remained very similar to those of “The American College of Rheumatology 1990 criteria[4]” that confer a high sensitivity and specificity, with 3 out of the following 5 criteria being required for diagnosis:

1. Age at onset > 50 years
2. New headache
3. Temporal artery abnormality
4. Elevated erythrocyte sedimentation ratio (ESR) of at least 50
5. An abnormal artery biopsy—with temporal artery biopsy (TAB) as the current gold standard test

The pathology of GCA stems from granulomatous inflammation of the walls of medium and large sized arteries, and particularly those of the external carotid arteries [5]. The vessel wall inflammation results in tissue ischemia in the distribution of the vessel’s blood supply, with the most dangerous consequences therefore being permanent *loss of vision* and large artery complications such as *aortic aneurysms and dissections*. Total or partial loss of vision is reported in up to 20% of people with GCA [6] but is rare once steroids have been started, whilst studies have shown 27% of GCA patients develop the aforementioned large artery complications [7]. In the long term, there is also a significant increased risk of associated cardiovascular disease [8] (including strokes and myocardial infarctions) and other complications including peripheral neuropathy, depression, deafness, and the side effects of the corticosteroid treatment itself.

The updated GCA guidelines from the National Institute of Health and Care Excellence (NICE) in September 2020 [9], highlight that the mainstay of treatment remains glucocorticoids. They state the following:

- If new visual loss or double vision are found, urgent ophthalmology review and treatment with intravenous methylprednisolone is required to prevent visual complications
- In the absence of new visual symptoms, immediate treatment with 40–60 mg prednisolone per day is recommended.

The subsequent overall duration of steroid treatment is variable, with a proportion noted by the guideline with chronic relapsing disease requiring “low doses of corticosteroids for several years” [9]. The side effects of steroids are well known and categorized in a variety of different diseases, and include weight gain, dyspepsia, muscle weakness, skin thinning, easy bruising, steroid-related diabetes, hypertension, bone loss, and increased risk of infection, as outlined in the NICE Clinical Knowledge Summary on corticosteroids [10].

Tocilizumab was approved by NICE in April 2018, for up to a year in relapsing or refractory GCA in combination with a tapering course of steroids; the US Food and Drug Administration (FDA) had authorized this 1 year prior in America in 2017. The main clinical evidence considered by the guidelines were from the GiACTA trial [11], a randomized control trial that identified a significant reduction in sustained glucocorticoid-free remission in GCA with a prednisolone taper, in those randomized to tocilizumab rather than a placebo. The NICE Technology appraisal guidance [TA518] from 2018 [12], specifically provides funding for use of tocilizumab in GCA in the UK when patients have *relapsing or refractory GCA disease, have not already had tocilizumab*, and if it is provided at the *agreed cost* with healthcare providers.

Tocilizumab itself is a monoclonal antibody against the interleukin-6 receptor (both membrane bound and soluble forms). It is already licensed as an immunosuppressant in other rheumatological diseases such as moderate to severe rheumatoid arthritis (RA) and systemic juvenile idiopathic arthritis (JIA) since the late 2000s, with data supporting its usage as safe and steroid sparing capabilities [13], with additional use in the Coronavirus-19 pandemic. Tocilizumab’s use in GCA therefore now offers an opportunity for broadened use of this already established drug, many years later.

## Case presentation

### Methods

This case series discusses 16 consecutive patients started on tocilizumab for relapsed or refractory GCA between August 2018 and April 2021, after the establishment of a new hub and spoke model. The central assessing hub was St George’s University Hospitals NHS Foundation Trust, a large tertiary rheumatology center in the south of England.

A new referral form for tocilizumab was utilized and all cases were discussed in a fortnightly multidisciplinary team meeting. Information was gathered for this analysis retrospectively through these referral forms and electronic patient records, with additional correspondence from referring clinicians and corroboration with patients themselves. The central dispensing pharmacy was at St George’s Hospital, who validated patient numbers and details.

A total of 16 patients were identified (Table 1): 11 female and 5 male, with an average age of 72.4 (range 61–82) years. Seven patients were internal referrals from clinicians at the tertiary center, with the remainder from local district general hospitals.

**Table 1:** Data collection from 16 patients with giant cell arteries started on tocilizumab

	Patient	A	B	C	D	E	F	G	H	I	J	K	L	M	N	O	P
	Age	80	80	76	76	79	82	75	72	63	61	72	75	59	76	70	63
	Sex	M	M	M	F	F	F	F	F	F	F	F	F	M	F	M	F
	Ethnicity	Other	Arabic	White	Other	White	White	Other	White	Other	White	White	White	White	White	White	White
	Duration of GCA disease (months) before TOC	5	11	6	25	71	61	1	12	30	7	22	148	52	9	24	24
Medication	Steroid dose at start of TOC (mg)	40	20	40	20	25	10	60	40	5	30	10	30	20	IM 10-weekly	20	20
	Most recent steroid dose (mg) and date	0, Aug 2019	5, Feb 2021	7, Feb 2021	0, Feb 2020	10, Jan 2021	2, Nov 2020	3, Jan 2021	10, Feb 2019	5, Dec 2020	10, May 2020	0, May 2020	5, May 2021	10, May 2021	–	7, March 2021	10, May 2021
	Months on TOC	13	18	22	25	12	12	31	12	17	1	17	13	8	15	1	12
	Concurrent DMARD dose at TOC start	MTX 15 mg qw	–	–	MTX 15 mg qw	–	–	–	–		–	MTX 25mg qw	–	Lef 20 mg od	–	–	MTX 25 mg qw
Blood results	ESR at presentation	28	49	15	32	55	13	51	60	110	10	63	40	–	8	38	–
	ESR before start of TOC	2	29	4	32	8.7	5	51	48	10	12	18	16	–	2	22	13
	ESR after start of TOC	2	4	2	20	2	2	42	23	6	2	5	2	–	2	2	–
	CRP at presentation	403	176	3.5	43	105	10.9	37	128	26	20	49	30	–	10	63	–
	CRP before start of TOC	7.8	9.1	< 1.0	5.8	3.3	5.1	37	18	< 4.0	8	7.2	4.5	–	< 4.0	35	20
	CRP after start of TOC	< 1.0	0.7	< 1.0	3.3	1.8	1.9	14	3.3	< 4.0	< 1.0	< 4.0	1	–	< 4.0	1.4	–
	Temporal artery biopsy	Neg	Pos	Pos	Neg	Pos	Inconclusive	Inconclusive	Pos	Neg	Neg	Neg-late	Not done	Neg	Not done	Neg	Pos
	Comorbidities		PMR, IHD, PVD	RA	PMR, HypoT	PMR	PMR, HTN	RA	CVA, vasculitis	Takayasu’s arteritis	HypoT		PMR		PMR		PMR

*GCA* giant cell arteritis, *TOC* tocilizumab, *DMARDs* disease modifying antirheumatic drugs, *MTX* methotrexate, *qw* once weekly, *ESR* erythrocyte sedimentation ratio, *CRP* C-reactive protein, *PMR* polymyalgia rheumatica, *IHD* ischemic heart disease, *RA* rheumatoid arthritis, *HTN* hypertension, *HypoT* hypothyroidism, *CVA* cerebrovascular accident, *PVD* peripheral vascular disease, *Neg* negative, *Pos* positive, *Lef* leflunomide

#### Tocilizumab and steroid dosing

The difference between daily prednisolone dosing before starting tocilizumab and the latest steroid dosing after treatment with tocilizumab for the 15 cases with this data, demonstrates a range of dose reduction from 0 to 57 mg, with a mean of 20.4 mg [95% CI 13.0–27.8 mg]. Three of the 15 cases were weaned from steroids completely.

#### Tocilizumab continuation and side effects

Five of the 16 cases stopped tocilizumab after the NICE guidance-funded 12 months, and an additional eight cases have continued the medication for longer by seeking access to additional funding.

Two cases (cases J and O) stopped tocilizumab completely—the former due to multifold side effects including epistaxis, deranged liver function tests, and a facial rash, and the latter due to the unusual circumstance of ocular syphilis manifesting. The causes for pausing or skipping doses of tocilizumab dosing are summarized below in Table 2, with 9 of the 16 cases affected.

**Table 2:** Summary of causes of pausing/stopping tocilizumab

Case	Duration of pause	Reason
B	1 month	Bruising as side effect
C	3 weeks	Concurrent cellulitis
D	1 week	Concurrent vomiting illness and bronchiectasis flare
G	3 months + 1 week	Hip replacement operation Low WCC and neutrophil count
H	1 month	Deranged liver function tests (LFTs)
J	Stopped after 1 month	Epistaxis, deranged LFTs, rash
M	2 weeks	Concurrent cellulitis
N	2 weeks	Trapped nerve operation
O	Stopped after 1 month	Ocular syphilis manifestation

#### Blood results and tocilizumab

**Fig. 1:**
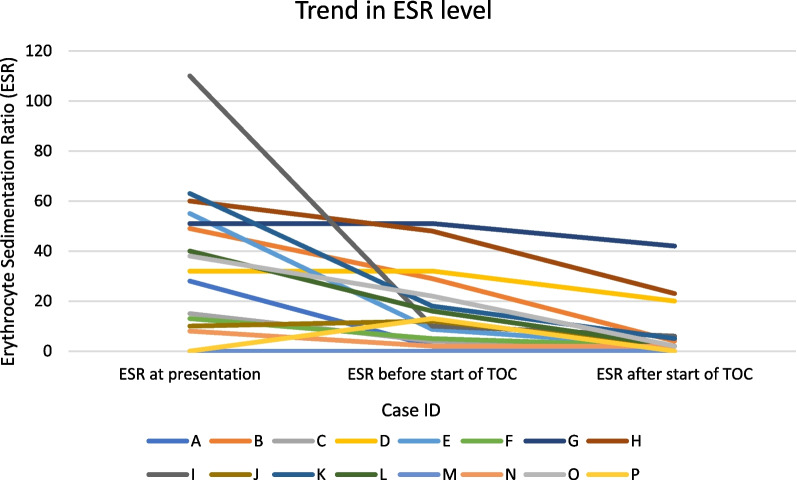
Trend in erythrocyte sedimentation ratio (ESR) level

Erythrocyte sedimentation ratio (ESR) (Fig. 1)

- The mean difference between ESR at presentation and before start of tocilizumab was 22.3 [95% CI 8.2–36.5].
- The mean difference between ESR before and after the start of tocilizumab was 10.3 [95% CI 5.8–14.7].

**Fig. 2:**
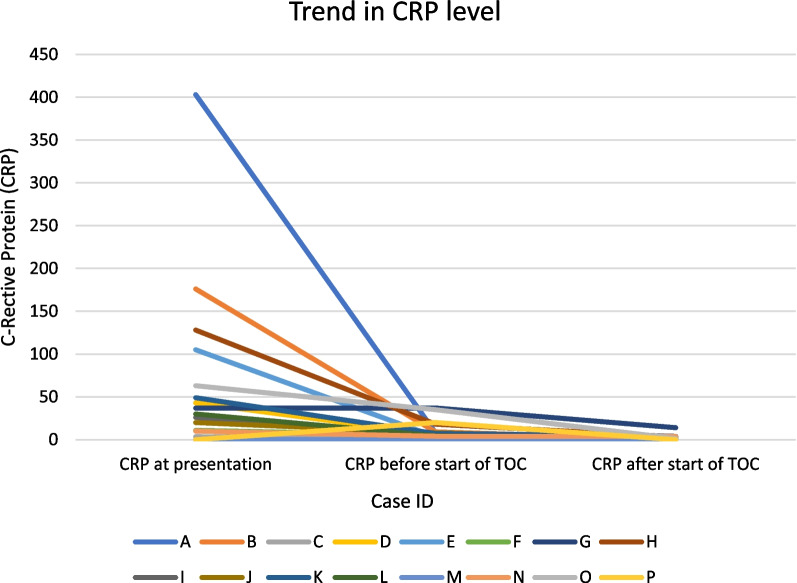
Trend in C-reactive protein level

C-reactive protein (CRP)—Fig. 2

- The mean difference between CRP at presentation and before start of tocilizumab was 68.2 [95% CI 12.5–123.8].
- The mean difference between CRP before and after the start of tocilizumab was 7.7 [95% CI 2.5–12.8].

Additional information about the cases are summarized in Table 3.

**Table 3:** Summary of additional data from 16 patients with giant cell arteries started on tocilizumab

Symptoms	Clinically, the most common symptoms on presentation were headache and jaw pain/claudication, each present in 75% of the patients (12 of 16 cases) Visual loss was present in 7 of the 16 cases, on a spectrum from blurry vision to complete visual loss in 3 individuals. Scalp tenderness was also present in 7 of the 16 cases Six cases reported weight loss, and three reported low appetite Six of the cases reported lethargy on presentation, and five reported sweats. Four described proximal myalgia More uncommon symptoms were reported in one to two cases each—including nausea, dizziness, early morning stiffness, and joint pain
Comorbidities	Seven of the 16 cases had a confirmed diagnosis of polymyalgia rheumatica, and two had existing rheumatoid arthritis. One had Takayasu’s arteritis—another large vessel vasculitis, and one had an additional unclassified vasculitis. The remaining five patients therefore had no additional primary rheumatological conditions
Investigations	Five of the 13 patients who had a temporal artery biopsy (TAB) were positive for GCA, with two samples inconclusive. Seven cases therefore had a negative biopsy, highlighting the established difficulties on reliance on TAB for diagnosis
Concurrent medication	Five cases were concurrently on methotrexate, with a dose range between 15 and 25 mg once weekly, and one additional case was on leflunomide as methotrexate was ineffective for them Thirteen of 16 cases were on vitamin D/calcium, 11 on alendronate, 9 on aspirin (with 1 further case on warfarin), and 15 on a gastroprotective agent (proton pump inhibitor/H2 receptor antagonist). Twenty-five percent were on a statin
Methylprednisolone	Fifty percent of cases required a 3-day intravenous methylprednisolone course, including all three of the cases with complete visual loss
Relapse	A relapse of GCA is defined as “the return of signs and/or symptoms with or without changes in inflammatory markers after reduction of therapy”[5], and this occurred for 13 of 16 cases. Five of these 13 cases had one relapse, four had two relapses, and the remaining four had multiple relapses (three or more)

## Discussions

### Referral pathway

The new hub and spoke clinical pathway established at this regional London center for the use of tocilizumab in relapsed and refractory GCA, facilitated the increased uptake of the newly licensed drug. This model has been already been well established at the hospital-level in the UK, with district general hospitals linked to centralized tertiary hospitals for treatment and specialist clinics [14], and here we have incorporated this organizational structure for the referral pathway. The referral pathway was simple to establish with the resources present, as it utilizes existing national health system (NHS) frameworks and systems, and the majority of patients referred to the pathway were accepted for tocilizumab therapy.

The efficacy of the pathway, however, relies on familiarity that the pathway itself exists by both registrars and consultants, knowledge of how to refer with the referral form required, and the administrative responsibilities of the centralized coordinating hub and pharmacy. The fortnightly multidisciplinary teams (MDTs) upon which the pathway hinged allowed for the additional opportunity for senior clinicians to discuss complex cases, and their experiences with and side effects of a relatively new treatment.

### Reduction in steroid burden

Analysis of 15 cases in this series demonstrates an average daily dose reduction of 20.4 mg prednisolone [95% CI 13.0–27.8 mg], and three cases were weaned from steroid treatment completely. This represents a considerable reduction in daily dosing and therefore overall steroid burden for those initiated on tocilizumab treatment. Current studies suggest that the average duration of treatment with steroids in GCA is 3 years, with a long steroid tapering period between 1 and 9 years [5]; while another study identified that only 24% of GCA patients were able to stop steroids within 2 years [15]. Studies in steroid use in polymyalgia rheumatica (PMR), GCA’s counterpart disease, have also highlighted that it is duration of steroid treatment rather than the initial prednisolone dose or maintenance dose, that contributes to greater side effects [16], signifying the impact of protracted courses of steroids and the danger of continuing courses for longer than necessary. This series therefore supports that a reduction and/or complete cessation of steroids is more possible with the addition of tocilizumab.

Consideration of the balancing measures mean it remains important however to highlight that glucocorticoids are and do remain the mainstay of acute management of GCA, as outlined in the introduction. Qualitative work has demonstrated that rheumatology clinicians are “often under pressure from patients and their primary care physicians to taper corticosteroids” and that this may not always be what is appropriate [17].

- If a patient has visual loss at any stage (that is both at presentation or whilst on steroid therapy as was the case for case G), intravenous methylprednisolone treatment must be instigated.
- Furthermore for patients who are on tocilizumab and concurrent steroids, steroid dosing should be increased to the previous higher dose if the patient is experiencing a relapse—as per EULAR guidelines [18]**.** This was commonplace in the series, with 13 of the 16 cases experiencing a relapse. However, this is not to say that a relapse precludes an eventual steroid wean whilst on tocilizumab—and case D demonstrates a scenario where a case who had two relapses was able to wean off steroids completely.

### Medications and steroid side effects

Both clinic letters and personal correspondence with referring clinicians highlighted that side effects from steroids were common and present in 11 of 16 cases, with weight gain in five cases and the serious osteoporotic complication of avascular necrosis in two cases. Existing larger prospective studies of steroid use in GCA and PMR have identified the most common side effects to be fractures, peptic ulcer perforations, and diabetes mellitus in this population cohort [19], and it is therefore important to try to rationalize steroids as much as is possible in an ageing population with existing comorbidities. Women are particularly affected by steroid side effects due to their hormonal and genetic predisposition to poorer bone health [20], and with GCA more common in women this is also pertinent to consider.

The concurrent prescription of gastroprotective medication with steroid use is well established, and the majority of our cases (15 out of 16) were on such an agent—13 on a proton pump inhibitor and a further 2 on ranitidine. This contrasted however to the medications for the reduction of cardiovascular risk, which were prescribed to a lesser extent—9 of 16 cases were on the recommended daily aspirin (with one additional case on warfarin), and only 25% were on a statin. This highlights room for improvement and a need for greater focus on the significant association of GCA with cardiovascular disease.

### Blood results

The inflammatory markers monitored were erythrocyte sedimentation ratio (ESR) and C-reactive protein (CRP), both relevant markers monitored in GCA [21], with ESR also forming part of the diagnostic criteria as outlined previously. All the cases showed a biochemical response with ESR and CRP, with differences in both markers after treatment with tocilizumab (respective means of 10.3 and 7.7). The greater difference, however, was the change in these markers at presentation and with treatment with steroids, with a mean ESR drop of 22.3 and CRP of 68.2.

The pathology of vasculitis is one of inflammation, and it is noted in the spectrum of patients suffering from GCA that those with a greater inflammatory response at diagnosis (looking at parameters of fever, weight loss, ESR) were more likely to have greater and longer steroid requirements [22]. Therefore, these markers may be more important at the stage of diagnosis of GCA and impact of initial steroid therapy, rather than with monitoring the disease due to the impact of tocilizumab, which itself blunts a CRP/ESR response.

### Tocilizumab side effects

Fourteen of the 16 cases completed 12 months treatment with tocilizumab, and out of the two who did not, case O had ocular syphilis and case J had multifold side effects. The longest duration of treatment break was 3 months (case G), due to a routine hip operation.

As the use of tocilizumab becomes more common, its side effects have been better characterized in literature. They are summarised below as per the Summary of Product Characteristics (SmPC) on the Electronic Medicines Compendium (EMC) [23], and we relate them to our case series in Table 4. The most commonly reported adverse drug reactions (ADRs) in the SmPC data are upper respiratory tract infections, nasopharyngitis, headache, and hypertension.

**Table 4:** Summary of side effects of tocilizumab from summary of product characteristics in relation to patients in our case series

Increased risk of infection—some studies have suggested a higher rate of serious infection with tocilizumab [24], specifically causing skin and subcutaneous infections [25], and complications of diverticulitis. The SmPC states to pause administration of tocilizumab until the infection is controlled	Case D and M both experienced cellulitis, that may have been independent or a side effect of tocilizumab. Both resolved with a pause in treatment of three and two doses, respectively
Liver function derangement—elevation in alanine aminotransferase (ALT) or aspartate aminotransferase (AST). If there is a change of greater than five times the upper limit of normal (ULN), the SmPC states to discontinue tocilizumab; if the change is between three and five times the ULN, to pausing dosing; and if the change is between one and five times the ULN, to dose modify	Liver function derangement was noted for both case H and J—case J stopped the treatment completely due to other causes, whilst case H paused treatment for 1 month, after which it resolved
Transient neutropenia—the SmPC states to not initiate tocilizumab if the neutrophil count is below 2 × 10^9^/L, to pause dosing if between 0.5 and 1 × 10^9^/L, and to stop if less than 0.5 × 10^9^/L	Case G skipped one dose due to a neutropenia
Thrombocytopenia—SmPC guidance states to discontinue tocilizumab if platelets are less than 50, and to pause dosing if between 50 and 100	Bruising was experienced by case B, with a 1 month pause in treatment
Elevation in lipid profile—including cholesterol, lipoproteins, and triglycerides. The SmPC suggests testing for these 1–2 months after initiation of tocilizumab, and starting appropriate hyperlipidemia management if required	
Hypersensitivity and infusion reactions—hypersensitivity is rare with subcutaneous tocilizumab [13], whereas infusion reactions are more common [25]	

These scenarios highlight the importance of close biochemical and clinical monitoring in those on this potent biologic. The consultant clinicians in the series approached tocilizumab prescribing with caution and care, and regular review and blood tests meant that dosing was very closely monitored. However, in the context of the new reliance of virtual care during the coronavirus pandemic, this level of monitoring is not always possible at present. The use of tocilizumab should therefore continue to be considered at a case-by-case level.

Further contraindications to tocilizumab include latent tuberculosis (TB) and hepatitis B and C, and all patients must be screened before starting tocilizumab [23]. If latent TB were to be identified, this must be fully treated before tocilizumab is started. Analysis of documentation in this case series identified avoidable delay between authorization of tocilizumab and initiation of treatment, due to the completion required for screening blood tests and chest radiographs for TB. Although not explicitly outlined in the guidance, other diseases can manifest upon initiation of tocilizumab, and case O was an unusual example of ocular syphilis being revealed and where treatment had to be stopped.

Of note, intravenous tocilizumab is to be stopped at least 4 weeks before any surgery, and subcutaneous tocilizumab at least 2 weeks before any surgery—as was the case for case G (hip replacement surgery) and N (operation for trapped nerve).

### Limitations

This case series is bound by the same limitations of any case series, including being retrospective and descriptive in nature. By having no control arm for the experiences of patients not taking tocilizumab, the conclusions formulated only suggest association and not causation. Selection bias was limited, however, by inclusion of all accepted MDT discussed cases within the specified time frame, and by corroborating cases with the pharmacy.

In regards to our data specifically, due to the nature of the hub and spoke referral pathway, it is perhaps understandable that a large proportion cases were referred from the hub hospital itself (7 of 16). Data from the spoke hospitals and referring district general hospitals were sometimes more limited due to not being electronic.

### What next?

Half of cases in this series continue tocilizumab treatment beyond the initial NICE guidelines limit of 12 months. Studies are currently being carried out to investigate this, including an extension to the original GiACTA trial—where patients not in full remission after a year were randomized to tocilizumab and/or glucocorticoids again [26]. This trial extension identified that re-treatment with tocilizumab was more associated with complete remission, and that overall glucocorticoid dosing over 3 years was lower in those who received tocilizumab rather than placebo in the first part of the trial. The NICE guidelines on tocilizumab in GCA are to be reviewed shortly, and we anticipate that the extension of the use of tocilizumab to be discussed.

## Conclusion

Our case series is the first published experience of the development and delivery of an effective hub and spoke referral pathway for tocilizumab treatment in GCA. We show that steroid dosing and duration can be reduced with tocilizumab, and that all subjects received full funding for treatment. Our referral pathway has encouraged the uptake of the IL-6 monoclonal antibody treatment for GCA and compliance with NICE guidelines. The drug offered improved disease control, and as more patients utilize the drug, seeks evidence to support ongoing funding for longer courses of tocilizumab.

## Data Availability

The data that support the findings of this study are available from the Institute for Infection & Immunity, St George’s University of London but restrictions apply to the availability of these data, which were used under license for the current study, and so are not publicly available. Data are however available from the authors upon reasonable request and with permission of St George’s University of London.

## Abbreviations

GCA: Giant cell arteritis
NICE: National Institute of Health and Care Excellence
FDA: Food and Drug Administration
TAB: Temporal artery biopsy
TOC: Tocilizumab
DMARDs: Disease modifying antirheumatic drugs
MTX: Methotrexate
qw: Once weekly
JIA: Juvenile idiopathic arthritis
ESR: Erythrocyte sedimentation ratio
CRP: C-reactive protein
PMR: Polymyalgia rheumatica
IHD: Ischemic heart disease
RA: Rheumatoid arthritis
HTN: Hypertension
HypoT: Hypothyroidism
CVA: Cerebrovascular accident
PVD: Peripheral vascular disease
Neg: Negative
Pos: Positive
Lef: Leflunomide
ALT: Aminotransferase
AST: Aspartate aminotransferase
ULN: Upper limit of normal
